# A Multi Clone Kinetic Model for characterizing Chinese hamster ovary cell line variability

**DOI:** 10.1093/jimb/kuaf029

**Published:** 2025-09-10

**Authors:** Devi Sietaram, Pavlos Kotidis, Gary Finka, Alexei A Lapkin

**Affiliations:** Department of Chemical Engineering and Biotechnology, University of Cambridge, Cambridge CB3 0AS, UK; BioPharm Process Research, GSK, Stevenage SG1 2NY, UK; BioPharm Process Research, GSK, Stevenage SG1 2NY, UK; Department of Chemical Engineering and Biotechnology, University of Cambridge, Cambridge CB3 0AS, UK

**Keywords:** cell line development, monoclonal antibodies, CHO cells, kinetic modelling, biotherapeutics

## Abstract

This paper presents the Multi Clone Kinetic Model (MCKM), a novel generalized kinetic mechanistic model for fed-batch cultivations of diverse Chinese hamster ovary (CHO) cell lines, producing different recombinant monoclonal antibodies (mAbs). Unlike traditional kinetic models requiring multiple cultures for one parameter regression, MCKM derives a complete set of 13 kinetic parameters from a single fed-batch cell line culture of 49 data points. This enables per-cell-line metabolic characterization during cell line development, as well as direct comparisons of kinetics across clones, passages, and different recombinant mAbs. To enable MCKM to be broadly applicable across many cell lines and mAbs, and to address the high-dimensional challenge of estimating 13 kinetic parameters from a small number of datapoints, the model uniquely incorporates a mechanistic growth constraint, a glucose-dependent lactate switch, and automated parameter balancing. MCKM demonstrated successful regression of 656 fed-batch culture runs of 157 unique CHO cell lines across four passage generations, recombinant for three different mAbs, achieving high accuracy in biomass and mAb titre (average ${\mathrm{\bar{R}}}_{{{{\mathrm{X}}}_{\mathrm{v}}}}^2$ ≈ 0.96 ± 0.07 and ${\mathrm{\bar{R}}}_{\mathrm{P}}^2$ ≈ 0.97 ± 0.05, respectively). MCKM could facilitate automated cell line selection, identification of critical process parameters and biomarkers, guide media and feeding strategies, predict metabolite profiles, and support scale-up and quality-by-design studies, delivering overall reduction of experimental workload.

**One-Sentence Summary:** This paper presents a novel kinetic model that derives distinct parameter sets from a single fed-batch run, enabling characterization of individual CHO clones across different mAb targets.

## Graphical Abstract

**Graphical Abstract:**
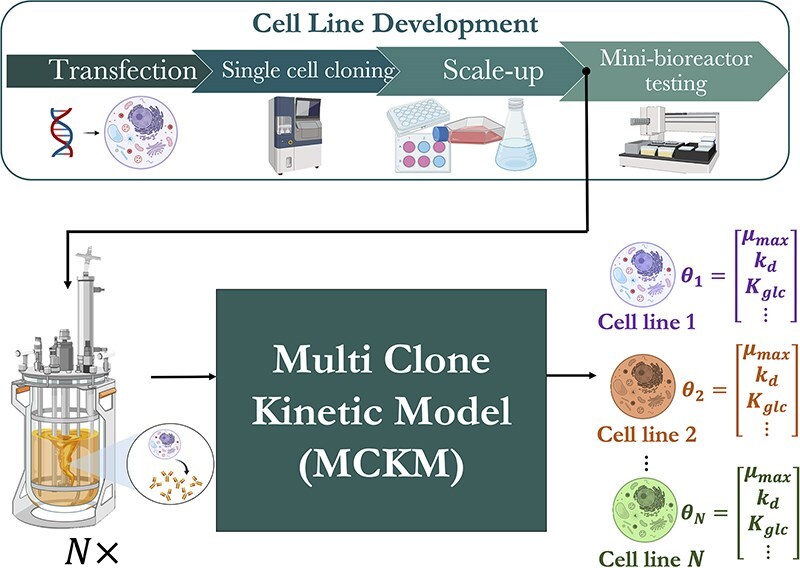

## Introduction

Monoclonal antibodies (mAb) are the largest growing class of biotherapeutics with global sales reaching $343 billion in 2021 (Walsh & Walsh, 2022). Chinese hamster ovary (CHO) cells have become the workhorse of industrial recombinant therapeutic protein production due to their flexibility in synthesising various proteins, human-like post-translational modifications, and adaptability to cell culture conditions (Kawabe et al., 2012; Pilbrough et al., 2009; Yang et al., 2015). Moreover, the Glutamine Synthetase (GS)-CHO cell line has gained prominence for its ability to produce complex biotherapeutics with high yields and product quality (Kim et al., 2012; Patel et al., 2018; Ye et al., 2009). However, CHO cells’ flexibility comes paired with its genomic plasticity, making the cells prone to genetic rearrangements and fluctuations in productivity over longer cultivations (Du et al., 2013; Kim et al., 2011). Consequently, after transfection, an upstream cell line screening study is required to identify cell lines that demonstrate consistently high productivity over multiple generations and cultivations, a process referred to as cell line development (CLD) (DeMaria et al., 2007).

CLD encompasses a series of experiments that aim to develop a clonal cell line with desired performance in production, and which commences with transfection. Transfection results in a heterogeneous pool of cells that differ in copy number and chromosomal positioning of the recombinant gene resulting from non-site-specific transfection and, therefore, leading to different growth and productivity behaviours (Reisinger et al., 2008). By cloning each cell into hundreds of clonal cell line cultures, the purpose of CLD is to assess interconal variation and to select the ‘lead’ clone or cell line, most appropriate for production in terms of growth, stable productivity and product quality. Cell line instability is still poorly understood; it introduces a stochastic level of heterogeneity between cell lines, which renders the process of identifying the lead cell line challenging (Kildegaard et al., 2013; Yusufi et al., 2017). The final stages of CLD consist of multiple rounds of fed-batch scale-down production runs of all cell lines, for example using the 15 mL microbioreactor (MBR) system Ambr15™ (Warr, 2020), aimed at selecting the cell line with adequate mAb productivity, product quality and cell line stability (i.e. stable productivity or product titre over >70 generations) (Dahodwala & Lee, 2019).

The optimization of the CLD screening and selection process would demand a deep understanding of the cellular kinetics governing cell line performance. Mechanistic modelling has been widely used in biochemical engineering to obtain a structured representation of knowledge about a cultivation process, developing fundamental understanding of the underlying biochemical mechanisms governing cell line behaviour (Costa et al., 2016). Among the various types of mechanistic modelling approaches, unstructured unsegregated kinetic models are most commonly used to capture the dynamic behaviour of cell lines, while assuming homogeneity in intracellular kinetics and cells (Kyriakopoulos et al., 2018). It is the hypothesis of this work that mechanistic modelling can provide a means to understand and predict the behaviour of the underlying processes and mechanisms of CHO cell culture. Furthermore, mechanistic modelling would allow for *in silico* simulation of fed-batch process, providing means to reduce the need for experiments. Mechanistic modelling of cell culture behaviour can be done through stoichiometric or kinetic modelling, while only kinetic modelling captures the dynamic behaviour of cell lines, which may be used in de-risking stability of cell culture scale-up, for example in Ambr15™ (Sha et al., 2018).

**Fig. 1.**
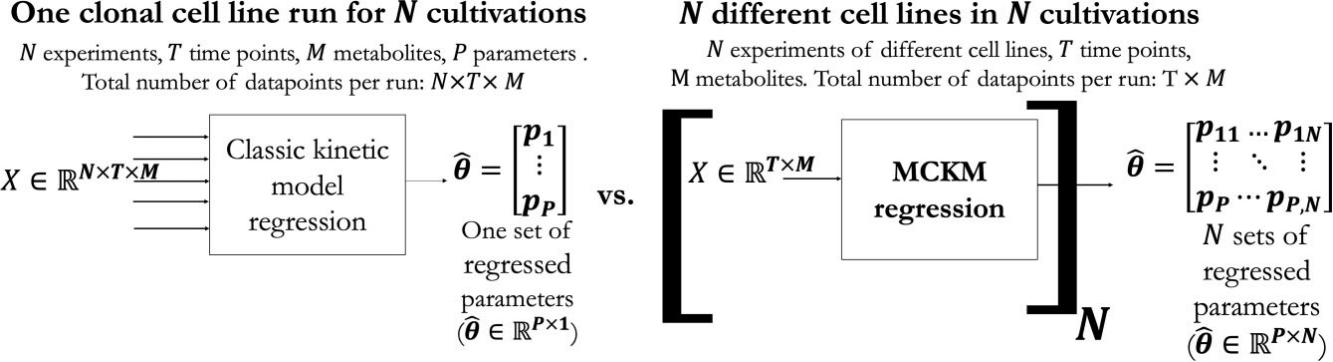
A comparison of conventional kinetic modelling that accumulates many cell cultures to regress one set of kinetic paraemters, versus the MCKM that uses a single cell line culture run to regress the kinetic parameters of each cell line culture run.

Developing a kinetic model for upstream CLD is challenging because it must be broadly applicable across different CHO host strains, clonal cell lines, and recombinant mAbs. Traditional kinetic models at the production scale focus on a single clone and mAb at a time, typically requiring multiple culture runs to generate a complete parameter set for one regression (Jang & Barford, 2000; López-Meza et al., 2016; Tang et al., 2020). This paper introduces a novel Multi Clone Kinetic Model (MCKM), which uniquely derives a set of 13 kinetic parameters from a single culture run for each CHO clonal cell line. Figure 1 illustrates how MCKM differs from conventional kinetic modelling, highlighting its ability to generate parameters for individual cell line runs. By characterizing each culture run in this way, the MCKM provides an additional layer of metabolic information from CLD data that can inform data-driven cell line selection.

The paper describes the MCKM model design in detail, and demonstrates its successful application to 656 fed-batch cell culture runs in the 15-mL minibioreactor Ambr15™. These data come from four historic CLD campaigns, encompassing 157 unique CHO cell lines, producing three distinct recombinant mAbs across two different CHO host strains. The paper also describes a sensitivity analysis and model identifiability study to evaluate the accuracy of the MCKM. Finally, the paper demonstrates an application of the MCKM to investigate metabolic differences among cell lines in relation to performance and stable productivity, using linear discriminant analysis (LDA).

## Methods

### Bioreactor Operation

The kinetic model was developed for fed-batch cultivation of recombinant CHO cells expressing high levels of mAb using the Glutamine Synthetase (GS) expression system. The data were recorded by GlaxoSmithKline, Stevenage, UK. The bioreactor system used was the Ambr15™ (TAP Biosystems, Greenville, DE) with a working volume of 15 mL. The data originate from four CLD campaigns where the different cell lines were all derived from the same CHO host. The campaigns are referred to as mAb-A, mAb-B1, mAb-B2, and mAb-C. These four campaigns involve three different mAbs, with mAb-B being campaigned twice using a different cell line platform (the exact difference is kept confidential). A total of 157 unique cell lines were studied, each progressing through four sequential Ambr runs to capture productivity data over at least 70 generations, enabling assessment of cell line stability. Cell line stability refers to consistent production across multiple passages or culture runs (Dahodwala & Lee, 2019). The four sequential runs are referred to as Ambr-1, Ambr-2, Ambr-3, and Ambr-4. Including some manually selected promising cell lines run in duplicates or triplicates, there are a total of 656 cell line culture runs (*N* = 656). Table 1 gives an overview of how many unique cell lines and cell cultures were run. Between each sequential Ambr run, cel lines are occasionally and manually selected and omitted based on poor cell line productivity, viability, or product quality, resulting in decreasing *N* over runs (exact selection criteria are kept confidential).

**Table 1.** An Overview of the Number of Cell Lines in Each Dataset and the Number of Cell Culture Runs (Batches)

	Ambr-1		Ambr-2		Ambr-3		Ambr-4	
	*N*	*n*	*N*	*n*	*N*	*n*	*N*	*n*
mAb-A	48	48	35	35	33	35	33	36
mAb-B1	19	20	18	18	18	18	18	20
mAb-B2	47	47	47	48	32	48	32	48
mAb-C	43	52	43	63	43	50	43	62

*N* is the number of unique cell lines, and *n* is the number of experiments, as sometimes particular cell lines are run in duplicate or triplicate. mAb-A, B1, B2, and C are the four CLD campaigns; Ambr-1, 2, 3, and 4 are the four sequential Ambr runs cultivating the same cell line over increasing generation numbers.

### Dataset

The total length of each Ambr15™ fed-batch cultivation is approximately 15 days. At-line samples were analysed on approximately day 0, 3, 6, 8, 10, 13, and 15, resulting in 7 (*T* = 7) samples in total, recording metabolic profiles of 7 metabolites (*M* = 7): viable cell concentration (VCC) (*X_υ_*), glucose (*glc*), glutamine (*gln*), glutamate (*glu*), mAb (*P*), ammonium (*amm*), lactate (*lac*), and for each Ambr15™ run. Furthermore, the fed-batch cultivations were run with bolus feeding, i.e. discrete feeding over time, of glucose and glutamate. Feeding of glucose and glutamate was executed right after sampling, with a total of five feeding times, commencing after the second sampling time. The feeding schedule of one individual run of mAb-A Ambr-1 was used as an approximation of the feeding of all other cell culture runs. Each cultivation is a dataset of seven-time measurements of the seven metabolites, resulting in a [7 × 7] matrix for each cell line culture run, as shown in Eq. 1.


(1) \begin{eqnarray*}
{{X}_{raw,\textit{data}}} = \left[ {\begin{array}{@{}*{1}{c}@{}} {{{X}_{v,t1}},{{P}_{t1}},gl{{c}_{t1}},gl{{u}_{t1}},gl{{n}_{t1}},am{{m}_{t1}},la{{c}_{t1}}}\\ \vdots \\ {{{X}_{v,t7}},{{P}_{t7}},gl{{c}_{t7}},gl{{u}_{t7}},gl{{n}_{t7}},am{{m}_{t7}},la{{c}_{t7}}} \end{array}} \right]
\end{eqnarray*}

#### Missing data

Some data were missing, such as day 2 lactate for mAb-A, Ambr-2, and day 1 protein titer for mAb-A, Ambr-2. For these samples, the kinetic model regression was run with fewer datapoints.

### Kinetic Model Development

Given the complexity of kinetic models—particularly in defining sufficient number of equations and parameters while avoiding overfitting—many mammalian cell bioprocess models have adopted a macroscopic approach (Chotteau et al., 2022). For the MCKM, the first step was to identify the key metabolic pathways governing growth and production, expressed through stoichiometric equations describing the interrelationships between major metabolites (Nolan & Lee, 2011). These were then translated into ordinary differential equations (ODEs) to capture temporal changes in metabolite concentrations. Mass balance expressions for production and consumption rates were formulated using established kinetics, including the Monod equation (Monod, 1949), derived from the Michaelis–Menten model (Michaelis & Menten, 1913), which has been widely favoured in describing enzyme kinetics due to its simplicity and ease of use for mathematical computations (Tang et al., 2020). For other metabolites, the Luedeking–Piret equation (Luedeking & Piret, 1959) was applied, relating production or consumption rates to growth rate and yield.

#### Biomass

The ODE of the viable biomass concentration (*X_v_* in 10^9^ cells L^−1^) is a function of the growth rate (*µ* in h^−1^) and death rate, Eq. 2.


(2) \begin{eqnarray*}
\frac{{d{{X}_v}}}{{dt}}{\mathrm{\ }} = \left( {\mu \left( t \right) - {{\mu }_d}\left( {\mathrm{t}} \right)} \right){\mathrm{*}}{{X}_v}\left( t \right)
\end{eqnarray*}

In kinetic models, growth is typically expressed as a function of limiting substrates such as glucose and glutamine, while toxic by-products (ammonium, lactate) may appear in the growth or death rate equations (Kornecki & Strube, 2019; López-Meza et al., 2016; Xing et al., 2010). In this study, glutamate was supplied in excess and thus not growth-limiting, while prior data analysis showed no significant correlation between VCC and ammonium or lactate in the growth phase (Sietaram et al., 2025). Consequently, glucose is the only growth-limiting substrate considered. The growth rate is described by Monod kinetics, as shown in Eq. 3.


(3) \begin{eqnarray*}
\mu \left( t \right) = {{\mu }_{max}}\frac{{glc\left( t \right)}}{{{{K}_{glc}}\ + \ glc\left( t \right)}},
\end{eqnarray*}

where *µ_max_* is the maximum growth rate (in h^−1^) and *K_glc_* (in mM) is the Michaelis–Menten constant of glucose or half-saturation concentration.

Previous data analysis (Sietaram et al., 2025) showed a significant negative Spearman correlation between VCC and ammonium during the death phase, suggesting ammonium-induced cell death. In line with the literature, where ammonium and lactate are both reported as cytotoxic by-products of protein metabolism (Karra et al., 2010), many CHO kinetic models include these metabolites in the death rate equation (Xing et al., 2010). However, the MCKM must remain robust across diverse behaviours, from clones that only produce lactate to those that switch from lactate production to consumption in the stationary phase (Carinhas et al., 2013; Gašperšič et al., 2018; Martínez et al., 2013). To ensure consistency, lactate was excluded, and the death rate equation was instead formulated as a Monod-type function of ammonium concentration alone, Eq. 4.


(4) \begin{eqnarray*}
{{\mu }_d}\left( {\mathrm{t}} \right) = {{{\mathrm{k}}}_{\mathrm{D}}}\frac{{amm\left( {\mathrm{t}} \right)}}{{K{{D}_{amm}}{\mathrm{\ }} + {\mathrm{\ }}amm\left( {\mathrm{t}} \right)}}
\end{eqnarray*}

where *k_D_* is the death rate (in h^−1^) and *KD_amm_* (in mM) is the Monod constant of ammonium inhibition.

#### Monoclonal antibody

The amount of secreted mAb in the extracellular environment (*P* in mM) formed over time is described by Eq. 5.


(5) \begin{eqnarray*}
\frac{{dP}}{{dt}}{\mathrm{\ }} = {\mathrm{\ }}{{q}_P}\left( {\mathrm{t}} \right){\mathrm{*}}{{X}_v}
\end{eqnarray*}

The form of the production rate for mAb (*q_P_* in mM h^−1^) can be described by the Luedeking–Piret model (López-Meza et al., 2016) as a product *Y_P/X_* (in mmol/10^9^ cells)—the yield of mAb, and biomass growth rate, Eq. 6.


(6) \begin{eqnarray*}
{{q}_P}\left( {\mathrm{t}} \right) = \mu \left( t \right){\mathrm{*}}{{Y}_{P/X}}
\end{eqnarray*}

#### Glucose

Glucose is the only growth-limiting substrate and is both being consumed, as well as being fed during the fed-batch cultivation, resulting in the ODE shown as Eq. 7.


(7) \begin{eqnarray*}
\frac{{\textit{dglc}}}{{dt}}{\mathrm{\ }} = - {\mathrm{\ }}{{q}_{glc}}\left( {\mathrm{t}} \right){\mathrm{*}}{{X}_v}\left( {\mathrm{t}} \right){\mathrm{\ }} + F\left( t \right){\mathrm{*}}\frac{{gl{{c}_{F,in}} - glc\left( {\mathrm{t}} \right)}}{{{{V}_L}\left( t \right)}},
\end{eqnarray*}

where *F(t)* is a discrete feeding function (in L h^−1^), representing the fed-batch bolus feeding, *glc_F,in_* (in mM) is the concentration of glucose in the feed, and *V_L_(t)* is the volume of the bioreactor (in L). The consumption rate of glucose (*q_glc_* in mM h^−1^) is described by the Luedeking–Piret model (López-Meza et al., 2016), Eq 8.


(8) \begin{eqnarray*}
{{q}_{glc}}\left( {\mathrm{t}} \right) = \frac{{\mu \left( t \right)}}{{{{Y}_{X/glc}}}} + {{m}_{glc}}
\end{eqnarray*}

where *Y_X/glc_* in (10^9^ cells mM^−1^) is the yield of biomass over glucose and *m_glc_* (in mmol L^−1^ h^−1^) is consumption of glucose for maintenance.

#### Glutamate

GS-CHO consumes glutamate as a substrate, where the majority of glutamate is converted into glutamine through the GS-system, as well as consumption for tricarboxylic acid (TCA) cycle intermediates or amino acids for protein synthesis (Zhang et al., 2006). Glutamate is fed throughout the cultivation, thus can be described as shown in Eq. 9.


(9) \begin{eqnarray*}
\frac{{d{\mathrm{glu}}}}{{dt}}{\mathrm{\ }} = - {\mathrm{\ }}{{q}_{glu}}\left( {\mathrm{t}} \right){\mathrm{*}}{{X}_v}\left( {\mathrm{t}} \right) + F\left( t \right){\mathrm{*}}\frac{{gl{{u}_{F,in}} - glu\left( t \right)}}{{{{V}_L}\left( t \right)}}{\mathrm{\ }}
\end{eqnarray*}

The consumption rate of glutamate (*q_glu_* in mM h^−1^) is described by the Luedeking–Piret model (López-Meza et al., 2016), Eq. 10.


(10) \begin{eqnarray*}
{{q}_{glu}}\left( {\mathrm{t}} \right) = \frac{{\mu \left( t \right)}}{{{{Y}_{X/glu}}}}
\end{eqnarray*}

#### Glutamine

Glutamine is mainly formed through the activity of GS that converts glutamate and ammonium into glutamine. Henceforth, glutamine production is a function of the glutamate consumption rate (*q_glu_*) with *Y_gln/glu_* (in mol mol^−1^) the yield of glutamine on glutamate. Furthermore, glutamine is consumed at a rate *q_gln_* for protein and biomass synthesis, Eq. 11.


(11) \begin{eqnarray*}
\frac{{\textit{dgln}}}{{dt}}\ = - {\mathrm{\ }}{{q}_{gln}}\left( {\mathrm{t}} \right){\mathrm{*}}{{{\mathrm{X}}}_{\mathrm{v}}}\left( {\mathrm{t}} \right){\mathrm{\ }} + {\mathrm{\ }}{{Y}_{\frac{{{\mathrm{gln}}}}{{{\mathrm{glu}}}}}}{\mathrm{*}}{{q}_{glu}}\left( {\mathrm{t}} \right){\mathrm{*}}{{{\mathrm{X}}}_{\mathrm{v}}}\left( t \right)
\end{eqnarray*}

The rate of consumption of glutamine (*q_gln_*) is described by the Luedeking–Piret model (López-Meza et al., 2016) as a function of yield *Y_X/gln_* (in 10^9^ cells mM^−1^), Eq. 12.


(12) \begin{eqnarray*}
{{q}_{gln}}\left( {\mathrm{t}} \right) = \frac{{\mu \left( t \right)}}{{{{Y}_{X/gln}}}}
\end{eqnarray*}

#### Ammonium

Ammonium (NH₃) is a by-product of cell growth and protein production. In GS-expression systems, glutamine synthase (GS) converts glutamate and ammonia into glutamine. The literature often links ammonium profiles to glutamine degradation (Xing et al., 2010), and for the GS-system specifically ammonium to be a function of glutamate (Kiparissides et al., 2015). Data analysis of the current dataset showed that ammonium correlates positively with glutamate only during the growth phase, and not in stationary or death phases (Sietaram et al., 2025). Most cell lines exhibited steadily increasing ammonium profiles, with occasional drops due to GS conversion. MCKM requires to accommodate the lack of a consistent link of ammonium to glutamate across all cell lines. The ammonium equation in MCKM is simplified to a general production term arising from aminoacid metabolism, Eq. 13.


(13) \begin{eqnarray*}
\frac{{d{\mathrm{amm}}}}{{dt}}{\mathrm{\ }} = {{q}_{amm}}\left( {\mathrm{t}} \right){\mathrm{*}}{{X}_v}\left( t \right)
\end{eqnarray*}

where the ammonium production rate (*q_amm_* in mM h^−1^) with Luedeking–Piret as a function of *Y_X/amm_* (in 10^9^ cells mM^−1^), Eq. 14.


(14) \begin{eqnarray*}
{{q}_{amm}}\left( {\mathrm{t}} \right) = \frac{{\mu \left( t \right)}}{{{{Y}_{X/amm}}}}
\end{eqnarray*}

#### Lactate

Lactate production is a result of the anaerobic conversion of glucose, where excess pyruvate is converted into lactate. This lactate production is often a function of the glucose consumption rate (Jang & Barford, 2000). A constraint could be used to introduce the lactate switch to the model, the transition from lactate production to consumption as cells enter the late exponential phase. The model must be flexible to be able to capture all cell lines behaviours where some cells exhibit and do not exhibit the switch to lactate consumption.

To the best of our knowledge, no kinetic model in the literature of CHO incorporates an accurate mathematical description of the lactate switch. The main identified cause for the lactate switch was glucose depletion (Hartley et al., 2018; Martínez et al., 2013). This was further validated in the current data set; data analysis has shown that lactate was found to significantly correlate to glucose profiles (Sietaram et al., 2025). Therefore, MCKM was designed such that the lactate switch depends on glucose depletion by incorporating an arbitrary minimum glucose concentration at which lactate consumption begins. This threshold was determined by analysing the 656 cell cultures and averaging glucose concentrations at which all cell lines started consuming lactate, resulting in a model constant *a_glc_* (in mM). This value is deduced only once from the data and further treated as a model constant. This is implemented into the kinetic model equation for lactate, as shown in Eqs. 15 and 16.


(15) \begin{eqnarray*}
\frac{{\textit{dlac}}}{{dt}} = {{q}_{lac}}\left( t \right)*{{X}_v}\left( t \right)
\end{eqnarray*}

(16) \begin{eqnarray*}
\left\{ {\begin{array}{@{}*{1}{c}@{}} {{{q}_{lac}}\left( {\mathrm{t}} \right) = {{{\mathrm{Y}}}_{\frac{{{\mathrm{lac}}}}{{{\mathrm{glc}}}}}}{\mathrm{*}}{{{\mathrm{q}}}_{{\mathrm{glc}}}}\ if{\mathrm{\ }}\left[ {glc} \right] < {{\alpha }_{glc}}}\\ {{{q}_{lac}}\left( {\mathrm{t}} \right) = - \frac{{\mathrm{\mu }}}{{{{Y}_{\frac{X}{{lac}}}}}}{\mathrm{*}}{{{\mathrm{X}}}_{\mathrm{v}}}\ if{\mathrm{\ }}\left[ {glc} \right] \ge {{\alpha }_{glc}}} \end{array}} \right.
\end{eqnarray*}

where *Y_lac/glc_* (in mol mol^−1^) is the yield of lactate on glucose, *q_glc_* is the glucose consumption rate, *q_lac_* (in mM h^−1^) is the lactate consumption rate, and *a_glc_* (in mM) is the concentration of glucose below which lactate consumption is activated.

### Model Implementation

#### Additional ODE for growth rate

The nature of the kinetic regression problem at hand is rather challenging. MCKM must estimate 13 kinetic parameters from a limited number of the available experimental data (49 datapoints) of a single culture run, while capturing the diverse behaviours of multiple CHO cell lines and mAbs. Thus, it is useful to introduce additional mechanistic constraints to attain the desired model accuracy. The unique feature of MCKM is the incorporation of an additional ODE describing the evolution of the growth rate (*µ*) over time. This acts as a mechanistic constraint, guiding regression towards parameter sets consistent with realistic growth dynamics. Prior to regression, the growth rate is estimated from raw VCC data (*µ_est_*, see the section ‘Expansion of input data with growth kinetics’) and treated as input alongside metabolite profiles. The model then regresses *µ_est_* against the growth rate ODE, which is derived using the quotient rule, Eq. 17.


(17) \begin{eqnarray*}
\frac{{d{{\mu }_{est}}}}{{dt}} = {{\mu }_{max}}*\frac{{\frac{{\textit{dglc}}}{{dt}}*{{K}_{glc}}}}{{{{{\left( {{{K}_{glc}} + glc} \right)}}^2}}}
\end{eqnarray*}

where *glc* is the glucose concentration (mM), *K_glc_* is the half-saturation constant of glucose (mM), *µ_max_* is the maximum growth rate (h^−1^) and *µ_est_* is the estimated growth rate from VCC data (h^−1^).

#### Bolus fed-batch model implementation

The kinetic model was implemented in Matlab^®^2019b as a set of ODEs. Since Matlab^®^2019b requires all variables, including time, to be continuous, the discrete nature of feeding poses a challenge. To address this, the fed-batch model equations were simplified into a batch kinetic model by removing the feeding terms. Fed-batch simulations are then run iteratively between feeding events, updating concentrations at each feeding step as new initial concentrations. The error of fed-batch regression is then calculated as a sum of errors of the iterative batch regressions. The seven ODEs as a batch model can be summarized as shown in Eq. 18.


(18) \begin{eqnarray*}
{{f}_{\textit{batch}}}\left( {t,{{X}_{\textit{initial}}},\theta } \right) = \left\{ {\begin{array}{@{}*{1}{c}@{}} {\frac{{d[{{X}_v}]}}{{dt}} = \left( {\mu - {{\mu }_d}} \right)*{{X}_v}}\\ {\frac{{d\left[ P \right]}}{{dt}} = \mu *{{{\mathrm{Y}}}_{P/X}}*{{X}_v}}\\ {\frac{{d\left[ {glc} \right]}}{{dt}} = - \left( {\frac{{\mathrm{\mu }}}{{{{Y}_{\frac{X}{{glc}}}}}} + {{{\mathrm{m}}}_{{\mathrm{glc}}}}} \right)*{{X}_v}}\\ {\frac{{d\left[ {gln} \right]}}{{dt}} = - \frac{{\mathrm{\mu }}}{{{{Y}_{X/gln}}}}*{{{\mathrm{X}}}_{\mathrm{v}}} + {\mathrm{\ }}{{Y}_{{\mathrm{gln}}/{\mathrm{glu}}}}*\frac{{\mathrm{\mu }}}{{{{Y}_{X/glu}}}}*{{X}_{\mathrm{v}}}}\\ {\frac{{d\left[ {glu} \right]}}{{dt}} = - \frac{{\mathrm{\mu }}}{{{{Y}_{\frac{X}{{glu}}}}}}*{{{\mathrm{X}}}_{\mathrm{v}}}}\\ {\frac{{d\left[ {amm} \right]}}{{dt}} = \frac{{\mathrm{\mu }}}{{{{Y}_{\frac{X}{{amm}}}}}}*{{X}_v}\ }\\ {\left\{ {\begin{array}{@{}*{1}{c}@{}} {\frac{{d\left[ {lac} \right]}}{{dt}} = {{{\mathrm{Y}}}_{\frac{{{\mathrm{lac}}}}{{{\mathrm{glc}}}}}}{\mathrm{*}}{{{\mathrm{q}}}_{{\mathrm{glc}}}}\ if{\mathrm{\ }}\left[ {glc} \right] < {{\alpha }_{glc}}}\\ {\frac{{d\left[ {lac} \right]}}{{dt}} = - \frac{{\mathrm{\mu }}}{{{{Y}_{\frac{X}{{lac}}}}}}{\mathrm{*}}{{{\mathrm{X}}}_{\mathrm{v}}}\ if{\mathrm{\ }}\left[ {glc} \right] \ge {{\alpha }_{glc}}} \end{array}} \right.\ }\\ {\frac{{d{{\mu }_{est}}}}{{dt}} = {{\mu }_{max}}*\frac{{\frac{{d\left[ {glc} \right]}}{{dt}}*{{K}_{\textit{Gglc}}}}}{{{{{\left( {{{K}_{glc}} + \left[ {glc} \right]} \right)}}^2}}}} \end{array}} \right.
\end{eqnarray*}

To run the bolus fed-batch kinetic model, the batch model is executed iteratively between the seven sampling times [*t_i_, t_i+1_*] resulting in six iterations. The model variables are *X(t)*=[*X_v_, P, glc, glu, gln, amm, lac, µ_est_*] and the kinetic parameters are:


\begin{eqnarray*}
\theta = \left[ {{{\mu }_{max}},{{k}_d},{{K}_{glc}},{{Y}_{\frac{X}{{glc}}}},{{Y}_{\frac{X}{{gln}}}},{{Y}_{\frac{X}{{glu}}}},{{Y}_{\frac{{lac}}{{glc}}}},{{Y}_{\frac{X}{{amm}}}},{{Y}_{\frac{{gln}}{{glu}}}},{{Y}_{\frac{P}{X}}},{{Y}_{\frac{X}{{lac}}}},{{m}_{glc}},{\mathrm{\ }}K{{D}_{amm}}} \right].
\end{eqnarray*}

The first batch run starts with the defined initial concentrations *X_initial_(t_0_)*. After each iteration, the *ode45* solver in Matlab^®^2019b computes *X_final_(t_i+1_)* between *t_i_* and *t_i+1_*. The discrete feeding *X_feeding_* (of substrates *glc* and *glu*) is added at *t_i+1_* to the *X_final_*, producing the new *X_initial_(t_i+1_)* for the next iteration. An overview of the bolus fed-batch model in pseudocode is given in Table 2.

**Table 2.** A Pseudocode of the Fed-Batch Bolus Feeding Model Implemented in Matlab as Iterations of a Batch Model *f_batch_* (Eq. 18)

1. Function run_fed_batch_bolus 1.1 *X_initial_* = [*VCC*_0_,*glc*_0_,*glu*_0_, *gln*_0_, *amm*_0_, *lac*_0_, *P*_0_, μ_*est*,0_] 1.2 *t_sample_* = [*t*_1_,*t*_2_,*t*_3_,*t*_4_,*t*_5_,*t*_6_,*t*_7_] 1.3 For iteration *i* = (*length*(*t_sample_*) − 1) 1.3.1 *t_initial_* = *t_sample_*(*i*) 1.3.2 *t_final_* = *t_sample_*(*i* + 1) 1.3.3 *X_final_* = *solve* *ode*45 *for* *f_batch_*(*t_initial_: t_final_,X_initial_*,θ) 1.3.4 *X_initial_* = *X_final_* + *X_feeding_*

### Parameter Regression

#### Expansion of input data with growth kinetics

The kinetic model is designed to regress a unique set of *θ* from one single cell culture run. In this study, a total of 656 (r = 656) Ambr15™ data cell line cultivations are regressed. To improve the accuracy of the model, an additional ODE for the estimated growth rate (*µ*) was incorporated into the model. No direct measurements of *µ* were recorded; hence the growth rate was rather estimated (*µ_est_*) from raw VCC data, derived from the assumption that the growth rate corresponds to the exponential of the VCC according to Goudar et al. (2008) and Xing et al. (2010), see Eq. 19.


(19) \begin{eqnarray*}
{{{\mathrm{\mu }}}_{{\mathrm{est}}}}\left( {{{{\mathrm{X}}}_{\mathrm{v}}},{{{\mathrm{t}}}_{\mathrm{i}}}} \right) = \frac{{\ln \left[ {{{{\mathrm{X}}}_{\mathrm{v}}}\left( {{{{\mathrm{t}}}_{{\mathrm{i}} + 1}}} \right)} \right] - \ln \left[ {{{{\mathrm{X}}}_{\mathrm{v}}}\left( {{{{\mathrm{t}}}_{\mathrm{i}}}} \right)} \right]}}{{{{t}_{i + 1}} - {{t}_i}}}
\end{eqnarray*}

Note that this assumes *X_v_* follows exponential growth at all times, which is an approximation, since growth may deviate from exponential behavior outside the true exponential phase.

For the first *µ_est,t1_* at *t_1_* the value calculated for *µ_est,t2_* is used twice. Thus, the raw input data *X_raw,data_* is expanded with *µ_est_*, resulting in *X_data_. X_data_* combines metabolite profiles with the growth rate estimates and is used as the actual input for regression; see *X_data_* described in Eq. 20.


(20) \begin{eqnarray*}
{{X}_{\textit{data}}} = \left[ {\begin{array}{@{}*{1}{c}@{}} {{{X}_{v,t1}},{{P}_{t1}},gl{{c}_{t1}},gl{{u}_{t1}},gl{{n}_{t1}},am{{m}_{t1}},la{{c}_{t1}},{{\mu }_{est,t1}}}\\ \vdots \\ {{{X}_{v,t7}},{{P}_{t7}},gl{{c}_{t7}},gl{{u}_{t7}},gl{{n}_{t7}},am{{m}_{t7}},la{{c}_{t7}},{{\mu }_{est,t7}}} \end{array}} \right]
\end{eqnarray*}

Prior to regression, *X_data_* was normalized into |*X_data_*| using *min-max scaling:*


(21) \begin{eqnarray*}
\left| {{{{{X}}}_{{{data}}}}} \right| = \frac{{{{{{X}}}_{{{data}}}} - \min \left( {{{{{X}}}_{{{data}}}}} \right)}}{{\max \left( {{{{{X}}}_{{{data}}}}} \right) - {{min}}\left( {{{{{X}}}_{{{data}}}}} \right)}}
\end{eqnarray*}

#### Objective function

The parameter regression was performed using least squared regression. Alternative methods such as Markov Chain Monte Carlo method or Bayesian optimization were considered impractical because the regression being performed on one cell culture run, as these optimization methods generally require more cell culture runs to perform parameter regression due to their inherent computational demands and the need for extensive sampling to ensure accuracy and convergence (Hernández Rodríguez et al., 2019; Ridgeway & Madigan, 2003; Xing et al., 2010).

The prediction variables are given as shown in Eq. 22.


(22) \begin{eqnarray*}
{{X}_{\textit{pred}}} = {{f}_{\textit{batch}}}\left( {{{t}_{\textit{data}}},|{{X}_{\textit{data}}}|\left( {{{t}_1}} \right),\theta } \right)
\end{eqnarray*}

The objective function *J* was defined as shown in Eq. 23.


(23) \begin{eqnarray*}
J\left( \theta \right) = \mathop \sum \limits_{i = 1}^{T - 1} {{{\mathrm{X}}}_{{\mathrm{pred}},{\mathrm{i}}}} - {{X}_{\textit{data},i}}
\end{eqnarray*}

*f_batch_(t_data_, X_data_(t_1_),θ*) (Eq. 18) returns *X_pred_*, predicted metabolite profiles, solved using *ode45* in Matlab^®^2019b. Due to the batch implementation of the kinetic model, the cost function is a summation in between time intervals to generate the fed-batch objective function. The *θ* parameters are regressed using the function *fmincon* and the algorithm *interior-point* (maximum number of function evaluations allowed 3E3) in Matlab^®^2019b according to:


(24) \begin{eqnarray*}
\mathop {\min }\limits_\theta J\left( \theta \right)\left\{ {{{\theta }_{lb}} < \theta < {{\theta }_{ub}}} \right.
\end{eqnarray*}

Global optimization methods were explored as an alternative to the interior-point algorithm, since the cost function is likely multi-modal with many local minima (Gábor et al., 2017; Miró et al., 2012). However, due to the nonlinear differential–algebraic equations and additional constraints, the optimization problem is ill-conditioned and highly sensitive to initial conditions. Small perturbations in input can cause large output variations, leading algorithms to converge to local optima or diverge (Sawicki et al., 2022; Villaverde et al., 2019). Moreover, global methods are computationally demanding, which is particularly problematic for such irregular optimization landscapes (Pal et al., 2005).

#### Initial and boundary conditions

This section describes how the initial guesses and bounds for the kinetic parameters, either mathematically or from the literature, were derived.

#### Initial guesses and bounds for yield

The initial guesses of the yields were estimated for each cell line were using the equation (Xing et al., 2010), see Eq. 25.


(25) \begin{eqnarray*}
{{Y}_{i/j}} = \left| {\frac{{{{c}_i}\left( {{{t}_7}} \right){\mathrm{*}}{{V}_L}\left( {{{t}_7}} \right) - {{c}_i}\left( {{{t}_1}} \right){\mathrm{*}}{{V}_L}\left( {{{t}_1}} \right)}}{{\left( {{{c}_i}\left( {{{t}_7}} \right){\mathrm{*}}{{V}_L}\left( {{{t}_7}} \right) - {{c}_i}\left( {{{t}_7}} \right){\mathrm{*}}{{V}_L}\left( {{{t}_7}} \right)} \right) + \mathop \sum \nolimits_{t = 1}^{t = 7} {{V}_{\textit{feed}}}{\mathrm{*}}{{c}_{i,\textit{feed}}}}}} \right|
\end{eqnarray*}

where *c_i_(t_1_), c_j_(t_1_), c_i_(t_7_)* and *c_j_(t_7_)* are the initial and the final concentrations of components *i* and *j*, respectively, *V_L_(t_1_)* and *V_L_(t_7_)* are the initial and the final volumes in the bioreactor, and *c_i,feed_* is the concentration of component *i* in the feed with a feeding volume *V_feed_*. When computing *Y_i/j_* for all cell culture runs, maximum and minimum values were used as the upper bounds (*θ_ub_*) and lower bounds (*θ_lb_*), respectively for parameter regression.

#### Initial guesses for µ_max_ and K_glc_

Prior to MCKM regression, a separate regression was performed only using concentration of *glc* and *µ_est_* data, to generate the initial guesses (IGs) for *µ_max_* and *K_glc_. µ_est_* was calculated from Eq. 19 and regressed against Eq. 3, assuming exponential growth of VCC. Only the first four time points were used here (*µ_est_, t_1_…µ_est_, t_4_*), corresponding to the strictly exponential growth phase (day 0–8). The sub dataset for this particular regression can be mathematically summarized as shown in Eq. 26.


(26) \begin{eqnarray*}
{{X}_{\textit{subdata}}} = \left[ {\begin{array}{@{}*{1}{c}@{}} {{{\mu }_{est,t1}},{\mathrm{\ }}gl{{c}_{t1}}}\\ \vdots \\ {{{\mu }_{est,t4}},gl{{c}_{t4}}} \end{array}} \right]
\end{eqnarray*}

Using the function *nlinfit* in Matlab^®^2019b, *X_subdata_* is non-linearly regressed upon the growth rate equation (Eq. 3). The regressed parameters are used as the initial guesses for *µ_max,IG_* and *K_glc,IG_*.

#### Remaining initial and boundary conditions

To define bounds and initial guesses of the remaining kinetic parameter, values from literature were used. The upper bounds for the yield kinetic parameters *Y_lac/glc_* and *Y_gln/glc_* were deduced from stoichiometry. All initial guesses and bounds used in the regression are given in Table 3.

**Table 3.** An Overview of Bounds and Initial Guesses of Kinetic Parameters

θ	unit	θ_*lb*_	θ_0_	θ_*ub*_	Literature	Reference
μ_*max*_	h^−1^	0.01	*	0.1	0.029–0.090	(López-Meza et al., 2016; Shakibaie et al., 2011; Xing et al., 2010)
*k_d_*	h^−1^	0.0001	0.016	0.1	0.016	(Xing et al., 2010)
*K_glc_*	g L^−1^	0.1	*	10	0.18–3.5	(Kiparissides et al., 2015; López-Meza et al., 2016; Shakibaie et al., 2011; Xing et al., 2010)
*m_glc_*	g L^−1^	0	0.006 192	1	0–0.036	(Xing et al., 2010)
*Y*_*X*/*glc*_	10^9^cells g^−1^	0.1	1**	10	0.42–2.8	(López-Meza et al., 2016; Shakibaie et al., 2011)
*Y*_*X*/*gln*_	10^9^cells mmol^−1^	0.1	0.974**	10	0.4–0.97	(Acosta et al., 2007; Xing et al., 2010)
*Y*_*X*/*glu*_	10^9^cells mmol^−1^	0.001	1**	20	-	-
*Y*_*lac*/*glc*_	g g^−1^	0.1	0.615	2***	0.5–0.615	(Martínez et al., 2013; Xing et al., 2010)
*Y*_*X*/*amm*_	10^9^cells mmol^−1^	0.1	0.67**	20	-	-
*Y*_*gln*/*glu*_	mol mol^−1^	0.001	0.7	1***	-	
*Y*_*X*/*lac*_	10^9^cells g^−1^	0.01	2.7**	5	0.89	(Shakibaie et al., 2011)
*Y*_*P*/*X*_	g/10^9^cells	0.0001	0.2**	1	-	-
*KD_amm_*	mM	0	5	100	1.44–14.28	(Kotidis et al., 2019a; Xing et al., 2010)

* computed for each cell line as described in ‘Initial guesses of *µ_max_* and *K_glc_*’

** derived from an average of the estimated yields as described in ‘Initial guesses yields’

*** derived from stoichiometry. For ‘-’ no values were found in the literature.

#### Model accuracy

For each regression or cell line, the goodness of fit was assessed through computing *R^2^* for each variable *i* in *X_pred_*, computed according to Eq. 27.


(27) \begin{eqnarray*}
R_i^2 = 1 - \frac{{\mathop \sum \nolimits_{i = 1}^n {{{({{X}_{\textit{data},i}} - {{X}_{\textit{pred},i}})}}^2}}}{{\mathop \sum \nolimits_{i = 1}^n {{{({{X}_{\textit{data},i}} - {{{\bar{X}}}_{\textit{data},i}})}}^2}\ }}
\end{eqnarray*}

where *X*_data_*_,i_* is the data of variable *i, n* is the number of datapoints (7) and ${{\bar{X}}_{\textit{data},i}}$ is the mean of the observed values of the *i*th variable. Furthermore, the normalized vector of the Root Mean Squared Error (RMSE) is computed as the % normalized RMSE (NMRSE) using min-max normalization (Eq. 21) upon |*X_data_*| and |*X_pred_*|:


(28) \begin{eqnarray*}
\textit{NMRSE} = \sqrt {{{{\left( {\left| {{{X}_{\textit{data}}}} \right| - \left| {{{X}_{\textit{pred}}}} \right|} \right)}}^2}} {\mathrm{*}}100{\mathrm{\% }}
\end{eqnarray*}

#### Automated parameter balancing

A common approach to model regression is parameter balancing, keeping some kinetic parameters fixed to reduce the parameter space to find optimal solutions (Lubitz et al., 2010).

**Fig. 2.**
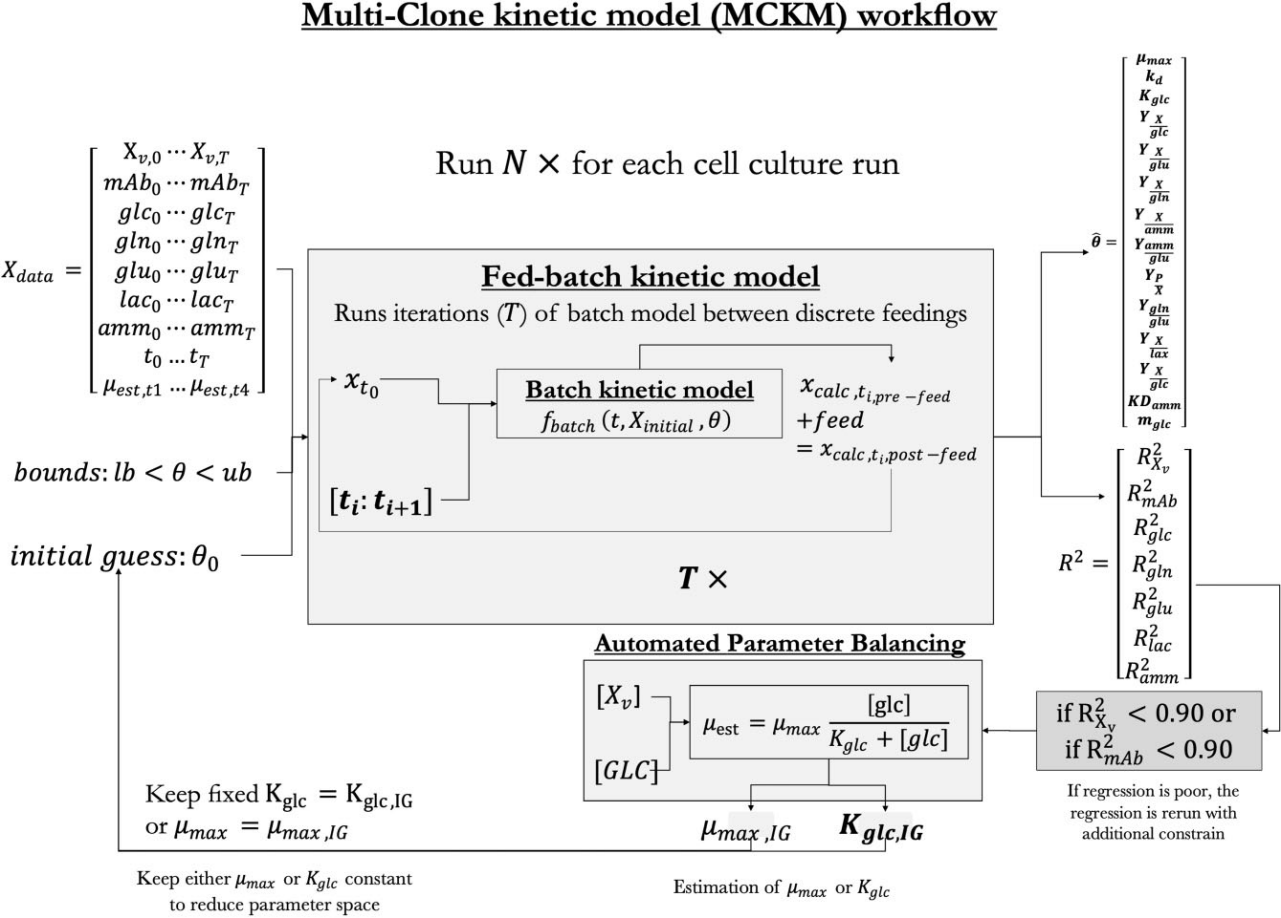
An overview of the Multi Clone Kinetic Model work flow. Each cell line culture run ***N ***regresses separately through the model. consisting of 49 datapoints (7 metabolites and 7 timepoints, additional constrains are implemented to result in a robust widely applicable model for many cell lines and different mAbs.

The kinetic model is now referred to as the ‘free-model’, comprising 13 kinetic parameters. During model development, potential co-linearity was identified between *µ_max_, k_d_* and *K_glc_* which can destabilize regression. In the literature, this is often addressed by fixing one or more parameters based on prior experimental measurements (Xing et al., 2010). However, such data were not available for all individual cell lines in this study. To address this, MCKM implements an *automated parameter balancing* procedure: if regression upon a certain cell line using the ‘free model’ fails, failing defined as $R_{{{X}_v}}^2$ <0.90 or $R_{mAb}^2$ <0.90, the parameter space is reduced to 12 instead of 13, keeping either *µ_max_* or *K_glc_* fixed. The values at which they are kept fixed are the initial guesses (*µ_max,IG_* and *K_glc,IG_*). Figure 2 shows an overview of the workflow of MCKM structure, and visualizes the automated parameter balancing.

### Model Identifiability

#### Sensitivity analysis

Whilst *R^2^* and *RMSE* assess accuracy of the kinetic model to fit the experimental metabolite profiles, parameter identifiability analysis is useful to confirm whether one unique solution for the set of kinetic parameters exists. This validation process ensures the biological significance of the regressed kinetic parameters, allowing the model to be utilized for metabolic analysis. A sensitivity analysis was executed for each of the parameters in *θ*). The sensitivity index (*s_ij_*) for each kinetic parameter *j* and each metabolite *i* was computed, averaging over the total number of timepoints (*T = 7*) was computed as shown in Eq. 29.


(29) \begin{eqnarray*}
{{\bar{s}}_{{\mathrm{ij}}}} = \frac{1}{{\mathrm{T}}}\mathop \sum \limits_{t = 1}^T \frac{{\partial {{X}_i}\left( t \right)}}{{\partial {{{\mathrm{\theta }}}_{\mathrm{j}}}}}{\mathrm{\ }}
\end{eqnarray*}

where *X_i_* is the observed variable or concentration of the *i*th metabolite. Averaging over all metabolites, the mean squared sensitivity of each parameter is given by Eq. 30 (Gábor et al., 2017).


(30) \begin{eqnarray*}
{\boldsymbol{\delta }}_{\boldsymbol{j}}^{{\boldsymbol{msqr}}} = \sqrt {\frac{1}{{\boldsymbol{n}}}\mathop \sum \limits_{{\boldsymbol{i}} = 1}^{{\boldsymbol{n\ }}} {\boldsymbol{s}}_{{\boldsymbol{ij}}}^2}
\end{eqnarray*}

#### Parameter identifiability

To assess if the parameters are uniquely identifiable, it was verified whether changes in parameters can be cancelled out by other parameters, due to collinearity. This is computed through the collinearity index (CI) between parameter pairs. The sensitivity index is first normalized:


(31) \begin{eqnarray*}
{{\bar{s}}_{ij}} = \frac{{{{s}_{ij}}}}{{\|{{s}_j}\|}}
\end{eqnarray*}

The normalized matrix $\bar{S} = \{ {{{{\bar{s}}}_{ij}}} \}$. The CI for each parameter pair *K* was computed as:


(32) \begin{eqnarray*}
C{{I}_K} = \frac{1}{{\surd \overline {{{{\bar{\lambda }}}_K}} }}
\end{eqnarray*}

where ${{\bar{\lambda }}_K}$ is the smallest eigenvalue of $\bar{S}_K^T{{\bar{S}}_K}$ (Brun et al., 2002) implemented using *eig()* in Matlab^®^2019b.

#### Biological interpretation of parameter estimates

The parameter regression was run a total of 656 times for each cell line culture run. The identified kinetic parameters were averaged for each of the four different mAbs campaigns separately, computing the mean values of the parameters across the cell lines and standard deviation which reflect the variance amongst cell lines. The identified kinetic parameter values were compared to those reported in the literature.

#### Case study: using the MCKM to identify key metabolic patterns in CLD

To demonstrate a biologically relevant application of the MCKM kinetic model, a case study is presented that uses the regressed kinetic parameters of the cell lines to study metabolic differences between well-performing and poorly performing, or stable and unstable cell lines. Well-performing cell lines are defined as scoring the top 50% mAb titre during Ambr-1, 2, 3, and 4, exhibiting minimal changes in productivity over time (i.e. low slope when productivity is plotted against generation) and maintaining a sufficiently high productivity level. Specific numerical thresholds were not disclosed due to confidentiality agreements. LDA, as proposed by (Fisher, 1936), was applied to the kinetic parameters acquired from both groups; well-performing versus poor-performing, or stable versus unstable cell lines. This quantifies the contribution of each kinetic parameter to both performance and stability.

## Results and Discussion

### Kinetic Model Regression

In this study, a generalized Multi Clone Kinetic Model (MCKM) was developed to regress kinetic parameters of individual CHO cell cultures producing different recombinant mAbs. The MCKM estimates 13 kinetic parameters per culture and was applied to 656 Ambr15™ fed-batch cultures spanning 157 unique CHO clonal cell lines, recombinant for three distinct mAbs. Table 4 contains the average accuracy of the 656 MCKM regressions in terms of *R^2^* and *RMSE* of the eight process variables, averaged overall all the cell lines that are recombinant for the same mAb as this data originates from four different CLD campaigns.

**Table 4.** MCKM Regression Performance Metrics of the Four Different CLD Campaigns

	mAb-A		mAb-B1		mAb-B2		mAb-C	
	Mean	Std	Mean	Value	Mean	Std	Mean	Std
$\bar{R}_{{{X}_v}}^2$	0.920	0.078	0.959	0.055	0.967	0.065	0.954	0.067
$\bar{R}_P^2$	0.935	0.045	0.970	0.023	0.975	0.057	0.985	0.043
$\bar{R}_{glc}^2$	-0.240	0.480	0.247	0.449	-0.041	0.374	0.246	0.520
$\bar{R}_{gln}^2$	0.671	0.501	0.888	0.124	0.866	0.208	0.885	0.169
$\bar{R}_{glu}^2$	0.355	0.354	0.508	0.359	0.271	0.331	0.336	0.537
$\bar{R}_{lac}^2$	0.206	1.984	-0.238	1.961	-0.220	3.455	-0.524	2.516
$\bar{R}_{amm}^2$	0.375	0.586	0.729	0.486	0.869	0.226	0.672	0.320
$NRMS{{E}_{{{X}_v}}}$	5.8%	3.9%	6.7%	3.1%	6.1%	4.3%	7.4%	0.035
*NRMSE_P_*	8.6%	3.9%	5.7%	2.3%	5.5%	3.9%	4.1%	0.033
*NRMSE_glc_*	46.3%	12.4%	49.7%	14.6%	50.6%	10.6%	50.4%	21.8%
*NRMSE_gln_*	15.8%	8.4%	10.9%	4.6%	12.9%	5.8%	10.3%	5.0%
*NRMSE_glu_*	34.2%	13.2%	33.8%	14.7%	39.8%	10.5%	35.1%	13.8%
*NRMSE_lac_*	33.5%	0.265	40.0%	21.1%	42.2%	28.0%	44.4%	22.0%
*NRMSE_amm_*	22.5%	10.9%	13.5%	10.4%	11.4%	6.8%	19.3%	7.9%

Tabulated are *R*^2^ and NRMSE values of regression of averaged over all cell lines of the same CLD campaign (mAb-A, B1, B2, or C). Values from different CLD campaigns are reported separately, as differences in mAb or platform can substantially influence cell line kinetics.

#### Modelling of growth and titre

Overall, MCKM demonstrated strong regression performance for biomass and mAb titre, with an average $\bar{R}_{{{X}_v}}^2 \approx $0.96 ± 0.07 and $\bar{R}_P^2 \approx $0.97 ± 0.05 (Table 4) averaged over the 656 regressions, indicating that the MCKM effectively captures diverse cell line behaviours. Out of the 656 parameter regressions, 99% runs are being predicted with ${{R}_{{{X}_v}}}$ > 0.90 and *R_P_* > 0.90. Compared to existing literature, the accuracy of the regression achieved in this study is comparable (Goudar et al., 2010; Kotidis et al., 2019b; López-Meza et al., 2016; Xing et al., 2010). However, it is important to note that MCKM operates very differently from the literature’s kinetic models, as it does not accumulate hundreds of cultures for one parameter regression but rather uses only one cell culture run (with 49 available data points in our case) to regress a set 13 kinetic parameters. Each regression of MCKM was performed independently, with no parameter sharing or transfer learning across experiments; the fitted parameters are specific to kinetics of each individual cell line culture run. Given this high dimensionality problem, MCKM’s performance can be considered remarkably robust. $NRMS{{E}_{{{X}_v}}}$ and *NRMSE_P_* of 6–7% and 4–9%, respectively outperforms hybrid modelling strategies and transfer models which range between 10% and 20% (Bayer et al., 2021).

**Fig. 3.**
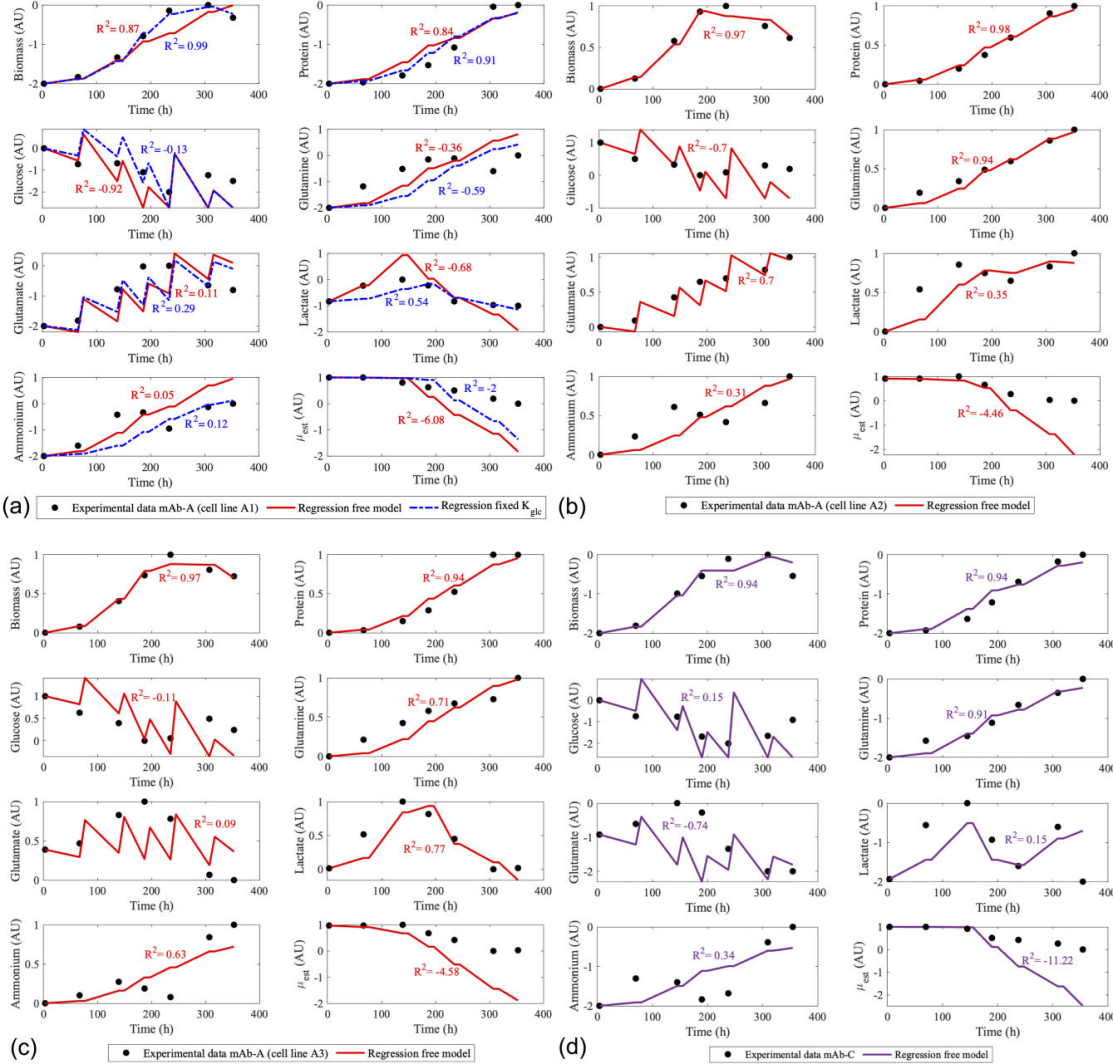
Regression results of the MCKM of four example cell line culture runs. To demonstrate a variety of cell line behaviours, plotted are (a) a cell line with significant lactate consumption (mab-A, Ambr-1, cell line A1); (b) a cell line with only increasing lactate (mab-A, Ambr-1, cell line A2); (c) a poorly growing cell line (mAb-A, Ambr-1, cell line A3), and (d) a cell line that fluctuates in lactate production to consumption (mAb-C, Ambr-1).

Three of the 656 MCKM cell line culture regressions are plotted in Fig. 3 to show a variety of cell line behaviours MCKM is able to capture. Figure 3a plots a cell line that was optimized using MCKM ‘automated parameter balancing’ described in the section ‘Initial guesses for *µ_max_* and *K_glc_*’. The initial ‘regression free model’ resulted in $R_{{{X}_v}}^2$ =0.87, which is below the threshold of accurate regression ($R_{{{X}_v}}^2$ <0.90). Consequently, MCKM automatically triggers its automated parameter balancing procedure, re-running the regression in a reduced parameter space of 12 instead of 13, keeping either *K_glc_* or *µ_max_* fixed. Figure 3a plots the ‘regression fixed *K_glc_*’ and shows improvement in biomass, protein, glucose, ammonium, and lactate regression compared to the ‘regression free model’, to $R_{{{X}_v}}^2$ = 0.99 and $R_P^2$ = 0.91. The other metabolites show only slight improvement, comparing ‘regression free model’ to ‘regression fixed *K_glc_*’, respectively, highlighting the model’s capacity to find multiple kinetic parameter solutions that still result it a similar plot of the metabolite profiles. This will be further analysed in the parameter identifiability study in the section ‘Parameter identifiability’.

Figure 3b shows a cell line with an earlier and steeper death phase, resulting in an overall reduced cell growth, which MCKM accurately captures with $R_{{{X}_v}}^2$ = 0.97 and $R_P^2$ = 0.98. Figure 3b and d also reveal that around the stationary phase (*t* = 250), MCKM predicts a dip in the VCC growth curve which is not observed in the experimental profiles. This arises because in MCKM death rate is defined as a function of ammonium, while ammonium is represented as a simple mathematical expression proportional to the growth rate. In contrast, the experimental data show a drop in ammonium around the stationary phase, see Figs. 3b and d, which MCKM regression cannot mathematically reproduce. Nevertheless, MCKM still predicts an appropriate peak of VCC. Peak value of VCC is often reported as a key metric in CLD, as it reflects the maximum viable biomass achieved during culture, and is frequently used in preliminary cell line assessments (Moore et al., 2024).

The glucose regressions in Fig. 3 visually demonstrate accurate fits of the profiles across the four cell lines. However, the coefficient of determination is negative and highly variable for glucose ($\bar{R}_{glc}^2$=-0.24 ± 0.48 and *NRMSE_glc_* ~50% for mAb-A in Table 4). Since MCKM applies average feeding amounts of glucose and glutamate across cell lines, while actual experiments involve slight variations in feeding amounts and timing, the statistical fit metric is sensitive to these discrepancies. This suggests that incorporating more precise feeding data or adopting a dynamic feeding model could further improve prediction accuracy for glucose and glutamate.

In all four cell line regressions in Fig. 3, *µ_est_* predicted by MCKM aligns well with the computed *µ_est_* from the experimental data during the exponential phase, but deviates in the stationary and death phases. This is because *µ_est_* is computed assuming exponential changes in VCC at all time points (Eq. 19). During the stationary and death phases, cells no longer follow strict exponential kinetics, leading to an inaccurate *µ_est_* in the stationary and death phase.

#### Modelling of the lactate switch

MCKM takes a mechanistic approach to modelling the lactate switch by introducing a minimum glucose threshold below which cells transition to lactate consumption. This successfully captures the characteristic parabolic lactate profiles of cell lines that have the lactate switch, see Fig. 3c, while also accommodates cell lines that lack the switch and solely produce lactate, see Fig. 3b. Figure 3d shows a cell line that activates the lactate switch twice, a fluctuation MCKM only partially captures. Table 4 shows a high average *NRMSE_lac_* of 33.5–44.4% across the four mAb campaigns. Nevertheless, despite its simplified formulation of the lactate switch, MCKM provides sufficient information to compare kinetic lactate profiles across different cell lines. Its strength lies not in absolute predictive accuracy, but in providing biologically interpretable parameters that allow comparison of lactate metabolism across cell lines—specifically, whether and how frequently cells undergo a lactate switch—while remaining computationally efficient and useful for CLD.

Future refinements in MCKM could link the switch to additional physiological drivers, such as redox balance or pH, to better capture complex fluctuations as the lactate switch is influenced by multiple factors, such as pH, NAD⁺/NADH ratio, mitochondrial function, and substrate availability (Hartley et al., 2018) as previous models have relied on data-driven or dynamic formulations (Villaverde et al., 2016; Zalai et al., 2015)

#### Modelling of the GS-metabolism

MCKM regressed well upon cell lines that are overall increasing in glutamate and ammonium, as shown in Figs. 3a and b, but does not capture drops in profiles glutamate and ammonium, as observed in Figs. 3c and d. This is also reflected in the low average *R^2^* in Table 4: $\bar{R}_{glu}^2$=0.355 ± 0.354 and $\bar{R}_{amm}^2$=0.375 ± 0.586 for mAb-A. This is a logical consequence of the simplification made in the ammonium equation, which does not mathematically link ammonium to glutamate in order to accommodate the wide variety of ammonium profiles across different cell lines. Additionally, MCKM uses an estimate of glutamate feeding, not the actual values that were used in the experiments, thus sensitivity of the *R²* metric to outliers contributes to these lower values (Chicco et al., 2021).

Notably, these drops in the glutamate and ammonium profiels are linked to the lactate switch as previous analysis found a significant correlation between lactate and glutamate in the death phase (Sietaram et al., 2025). Glucose limitation during this phase drives higher lactate and glutamate consumption, with glutamate potentially influencing lactate production via its role in the TCA cycle and amino acid biosynthesis (Széliová et al., 2020). This indicates an area for further refinement, such as integrating additional metabolic pathways or dynamic feedback mechanisms linking glutamate and ammonium consumption to glucose depletion. MCKM simplifies ammonium expression by not explicitly linking it to glutamate or glutamine, even though in reality this occurs via the GS system. This simplification was necessary because MCKM needed to accommodate a wide variety of cell lines and profiles, as revealed by data analysis the correlation between glutamate and ammonium is not consistent across cell lines (Sietaram et al., 2025).

The regression coefficient for glutamine differs notably between mAb-A and the other mAbs (Table 4). For mAb-A, the average $\bar{R}_{gln}^2$=0.671 ± 0.501, substantially lower than the values observed for the other three campaigns ($\bar{R}_{gln}^2$> 0.8, Table 4). The *NRMSE_amm_* of mAb is also higher (22.5%) than the other CLD campaigns (13.5 and 11.4% of mAb-B1, B2 Table 4). This suggests greater metabolic variability in glutamine utilization by the CHO cells producing mAb-A. The broad variance of the $\bar{R}_{gln}^2$ (std ± 0.501 for mAb-A) further indicates instability or inconsistency in how glutamine impacts production. In contrast, the higher and more consistent *R²* values for the other mAbs may reflect a tighter regulation of glutamine metabolism and a closer coupling between glutamine availability and protein synthesis (Goudar et al., 2010; Jeong et al., 2016). *NRMSE_gln_* and *NRMSE_amm_* in Table 4 are 11–22%, which is a reasonable level of error compared to the literature, given the simplified equation of MCKM for these metabolites.

### Model Identifiability

When using MCKM predictions to study kinetics across cell lines in CLD, their biological interpretability is crucial. To assess this, model identifiability is evaluated—determining whether the model parameters can be accurately and uniquely inferred from the input-output data (Sidoli et al., 2003). This involves performing a sensitivity analysis, followed by quantifying the co-linearity between parameters.

#### Sensitivity analysis

**Fig. 4.**
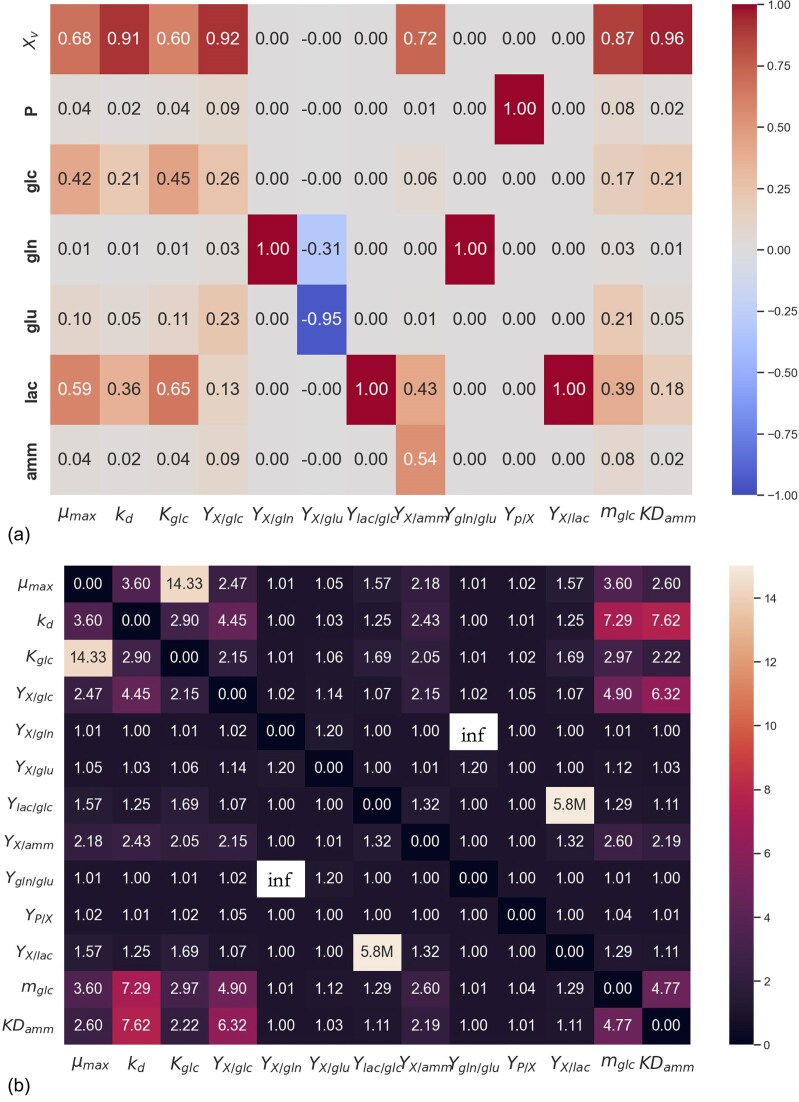
Parameter identifiability study: (a) normalized sensitivity coefficients, (b) collinearity index. M stands for million. The plot (a) shows normalized standard sensitivity coefficients, where positive values indicate that increasing the parameter increases the model output, and negative values indicate an inverse effect. Values near zero suggest low sensitivity. The collinearity index in (b) quantifies the degree of parameter correlation, with higher values indicating potential identifiability issues due to parameter interdependencies.

To assess model identifiability, a sensitivity analysis was first performed to evaluate how variables in the kinetic model respond to the 13 kinetic parameters. Figure 4a shows normalized sensitivity coefficients. As can be seen, MCKM process variables exhibit sensitivity to all 13 kinetic parameters. Biomass is naturally sensitive to all the parameters that occur in the growth rate equation (*µ_max_, K_glc_, m_glc_*) and death rate equation (*k_D_* and *KD_amm_*). The kinetic model characterizes cell growth, with glucose serving as the sole growth-limiting substrate and ammonium as the sole growth inhibitor, resulting in a normalized sensitivity coefficient of *X_v_* to *Y_X/glc_* of 0.92 and *Y_X/amm_* of 0.72, see Fig. 4a.

The substrates glucose and glutamate in Fig. 4a both show sensitivity to growth and death rate kinetic parameters (*µ_max_, K_glc_, m_glc_, k_D_* and *KD_amm_*), which is expected, since substrate availability directly affects cell proliferation and death. Notably, the growth model is highly sensitive to *KD_amm_* (normalized sensitivity of 0.96, Fig. 4a). Lactate is also significantly sensitive to *µ_max_* (normalized sensitivity of 0.59) and *k_D_* (normalized sensitivity of 0.36) parameters, see Fig. 4a, highlighting that lactate production and consumption are closely tied to the cells’ metabolic state. While the model captures lactate as a consequence of growth and death, the causal relationship may be bidirectional: lactate could also influence or induce cell death, a potential interaction not currently included in MCKM to maintain generality across diverse cell line lactate profiles. Additionally, lactate shows sensitivity to *Y_X/amm_* (normalized sensitivity of 0.43, Fig. 4a), reflecting MCKM's mathematical link between ammonium and death rate, and the biological association between lactate accumulation and cell death.

#### Parameter identifiability

To assess if all parameters are uniquely identifiable, the co-linearity index was computed from the normalized sensitivity index (through Eq. 28–30), of which the computed values are shown in Fig. 4b. A co-linearity index of CI_k_ > 20 is considered co-linear where roughly 95% of the variation in the model output by changing one parameter can be compensated by the other parameter (Gábor et al., 2017). The co-linearity index between *µ_max_* and *K_glc_* is CI_k_ = 14.33, implying a slight co-linearity but still below the threshold. This co-linearity arises because both parameters influence the rate of growth and are often interdependent in their effects on cell growth kinetics. Conceptually, *K_glc_* describes the cell’s affinity for glucose, which naturally impacts the maximum rate at which the cells can grow. The kinetic model has the automated parameter balacning for regression runs that fixies either *µ_max_* or *K_glc_* based on *µ_max,IG_* or *K_glc,IG_*, respectively, in which the co-linearity is directly overcome.

*m_glc_* and *KD_amm_* also demonstrate some co-linearity, with the growth parameters *µ_max_* or *K_glc_* and *k_D_*, although the CI_k_ values remain below the identifiability threshold. This mild co-dependence reflects the underlying biology, as both basal glucose consumption and ammonium-induced cell death are inherently linked to growth dynamics.

A high degree of collinearity is observed between *Y_lac/glc_* and *Y_X/lac_* (CIK = 5.8×10^6^, see Fig. 4b). The structure of the ODE for lactate (equation) naturally leads to collinearity between *Y_lac/glc_* and *Y_X/lac_*as both parameters govern the same underlying biological process. How much of the cell's glucose is consumed for lactate (*Y_lac/glc_*) naturally impacts the yield of biomass upon lactate consumption; in other words, *Y_X/lac_* is biologically a function of *Y_lac/glc_*. It is common for kinetic models of biochemical systems to be practically unidentifiable, as there is a complex interplay between biological processes that these parameters represent, making it difficult to decouple their effects (Gábor et al., 2017). While *Y_lac/glc_* and *Y_X/lac_* cannot be uniquely determined, MCKM reliably captures lactate profiles, such as if and when the cells switch from lactate consumption to production, allowing comparison of metabolic switching and lactate utilization of patterns between cell lines.

The same principle applies to *Y_gln/glu_* and *Y_X/gln_*, which are co-linear (CI_k_ = inf, Fig. 4b). While fixing one of the co-linear parameters is necessary to achieve unique parameter estimates, caution is needed when interpreting the absolute parameter values, as they may not fully represent the true metabolism. However, relative differences in glutamine consumption and conversion between cell lines can still be assessed, providing useful information for evaluating cell line metabolic behavior.

### Parameter Estimates of Different Cell Lines

The regressed kinetic parameters were averaged across all cell lines producing of the mAb CLD campaign, with mean values and standard deviations reported in Table 5. The standard deviation reflects variability amongst the cell lines of he same mAb campaign. For all four mAbs, the maximum growth rate *µ_max_* ≈ 0.02 h^−1^ (Table 5) is on the lower end of the literature values for recombinant CHO: *µ_max_* ≈ 0.029–0.090 h^−1^ (Aehle et al., 2010; Kotidis et al., 2019a; López-Meza et al., 2016; Shakibaie et al., 2011; Xing et al., 2010). The low standard deviation in *µ_max_* (±0.00831 for mAb-A amongst cell lines, Table 5) suggests this is an intrinsic property of the CHO host rather than a modelling artefact. From the computations of estimates of the growth rates, *µ_est_* (Eq. 19), the highest observed value was 0.0238 h^−1^, which aligns well with the regressed *µ_max_* ≈ 0.0258 ± 0.00831 h^−1^, supporting the plausibility of the accurate computation of *µ_max_* through MCKM.

**Table 5.** Mean Estimated Kinetic Parameters for All Cell Lines of the Four CLD Campaigns mAb-A, B1, B2, and C Ambr-1

		mAb-A		mAb-B1		mAb-B2		mAb-C	
θ	Unit	Mean*	Std**	Mean*	Std	Mean*	Std	Mean*	Std
μ_*max*_	h^−1^	0.0258	± 0.00 831	0.0221	± 0.0174	0.0237	± 0.00 390	0.0237	± 0.00912
*k_d_*	h^−1^	0.0180	± 0.0194	0.0166	± 0.0146	0.0176	± 0.0170	0.0185	± 0.0163
*K_glc_*	g L^−1^	1.05	± 2.60	0.788	± 3.263	0.501	± 1.35	0.641	± 2.67
*Y*_*X*/*glc*_	10^9^ cells g^−1^	2.03	± 1.29	1.52	± 2.028	2.46	± 2.13	2.45	± 1.97
*Y*_*X*/*gln*_	10^9^cells mmol^−1^	43.3	± 21.9	37.68	± 24.71	25.82	± 22.2	30.03	± 27.3
*Y*_*X*/*glu*_	10^9^cells mmol^−1^	8.94	± 4.19	7.680	± 4.774	7.95	± 3.65	8.828	± 5.49
*Y*_*X*/*amm*_	10^9^cells mmol^−1^	11.0	± 4.71	8.679	± 5.232	9.51	± 4.54	9.88	± 5.62
*Y*_*lac*/*glc*_	g_lac_/g_glc_	0.286	± 0.158	0.2684	± 0.1610	0.225	± 0.151	0.168	± 0.135
*Y*_*gln*/*glu*_	mol_gln_/mol_glu_	0.387	± 0.165	0.5227	± 0.2623	0.482	± 0.265	0.715	± 0.260
*Y*_*X*/*lac*_	(10^9^cells)/g_lac_	3.16	± 5.37	3.295	± 4.646	4.37	± 4.32	4.07	± 5.09
*Y*_*P*/*X*_	g_P_/(10^9^cells)	0.0481	± 0.0258	0.0898	± 0.0495	0.0561	± 0.0356	0.0892	± 0.0587
*m_glc_*	$\frac{{\mathrm{g}}}{{{{{10}}^9}{\mathrm{cells}}}}/{\mathrm{h}}$	1.51*10^−6^	2.07*10^−3^	1.60*10^−4^	± 2.92*10^−3^	1.71*10^−5^	± 1.48*10^−3^	8.80*10^−4^	± 3.07*10^−3^
*KD_amm_*	mM	6.47	± 3.14	6.4640	± 3.3144	4.17	± 3.30	6.29	± 3.42

Values from different CLD campaigns are reported separately, as differences in mAb or platform can substantially influence cell line kinetics. *These values are the average over the ~20–50 cell lines of the same mAb campaign in Ambr-1. **Std reflects the variation amongst cell lines.

MCKM finds average death rates of *k_d_* = 0.0116–0.0185 h^−1^ (Table 5) which match literature reports of 0.016 h^−1^ (Xing et al., 2010). Similarly, the biomass yield on glucose *Y_X/glc_* (≈2 × 10^9^ cells/g_glc_) and the glucose saturation constant *K_glc_* (≈1.05 g L^−1^ for mAb-A) fall within the reported ranges in the literature (Kiparissides et al., 2015; López-Meza et al., 2016). However, *K_glc_* shows a substantial variability between cell lines (standard deviation of ± 2.60 g L^−1^ for mAb-A). Considering the unstable CHO karyotype as a result of common chromosomal aberrations, random gene expression fluctuations, genomic rearrangements, gene copy number loss variance in cell culture conditions and cell line adaptation, a high variance in glucose metabolism amongst cell lines is plausible (Dahodwala & Lee, 2019; Lai et al., 2013).

Glutamine-related parameters, ${{\bar{Y}}_{X/gln}}$ and ${{\bar{Y}}_{X/glu}}$, exhibit larger variability (e.g. ${{\bar{Y}}_{X/gln}}$ = 43.3 ± 21.9 × 10^9^ cells mM^−1^ for mAb-A) primarily due to co-linearity in the model and the nature of GS-CHO cultures. From the currently measured experimental data, the kinetic model has no exact way of deriving how much glutamine is consumed as there is always a net increase of glutamine from the GS-conversion and glutamine is not a limiting nutrient, which is common in GS-CHO culture (Xu et al., 2014). The large variability in ${{\bar{Y}}_{X/glu}}$ (8.94± 4.19 × 10^9^ cells mM^−1^ for mAb-A) may reflect the large differences in GS-expression amongst cell lines as a result of random site of integration of the recombinant gene as well as gene copy number variation (Kuo et al., 2018; Würtele et al., 2003).

The lactate yields *Y_lac/glc_* and *Y_X/lac_* were found to be co-linear in the section ‘Parameter identifiability’, yet *Y_lac/glc_* remains relatively stable (0.286 ± 0.158 *g_lac_/g_glc_* for mAb-A, see Table 5), indicating consistent glucose partitioning to lactate across cell lines of the same mAb-campaign. This likely reflects the controlled microenvironment in Ambr15™ systems, which provide good control over dissolved oxygen levels, which can stabilize the reliance on anaerobic pathways (Kelly et al., 2018; Martínez et al., 2013; Xing et al., 2010).

Finally, the ammonium death rate constant *KD_amm_* is consistent across CLD the mAb-A, B1, and C campaigns and cell lines around ~6 ± 3 mM (Table 5). This is slightly higher than literature values (1.44–4.5 mM; Xing et al., 2010), likely due to model simplifications where cell death depends solely on ammonium concentration, omitting other potential stressors such as nutrient depletion or hypoxia (Karra et al., 2010). The results obtained with the MCKM model demonstrate how kinetic parameters provide insight into comparison of CLD campaigns, highlighting an altered ammonium metabolism for mAb-B1 (~4.2 ± 3 mM compared to 6 mM for the other three campaigns).

The mean *m_glc_* regressed by the kinetic model (1.51*10^−6^± 2.07*10^−3^  $\frac{{\mathrm{g}}}{{{{{10}}^9}{\mathrm{cells}}}}/{\mathrm{h}}$ for mAb-A, Table 5) varies significantly amongst cell lines and is lower than values observed in the literature of 0.036 $\frac{{\mathrm{g}}}{{{{{10}}^9}{\mathrm{cells}}}}/{\mathrm{h\ }}$(Xing et al., 2010) as a result of the parameter co-linearity (as was shown in the section ‘Parameter identifiability’). Thus, this value cannot be accurately deduced by the MCKM alone and would require complementary experimental measurements of basal glucose uptake to be reliably estimated.

### Case study: Using MCKM to Identify Key Metabolic Patterns in CLD

This case study demonstrates one example of how MCKM can be used to study metabolic differences amongst cell lines during CLD, namely to understand the biological mechanisms that underpin cell line stability, something that is currently poorly understood in the literature (Yusufi et al., 2017). Beyond this, MCKM could be leveraged in several other ways: for biomarker discovery to link metabolic parameters to productivity or stress response, for media and feeding strategy optimization, or in combination with machine learning approaches to predict high-performing clones or anticipate metabolic bottlenecks. In this study, we demonstrate a LDA applied to the regressed kinetic parameters, distinguishing well- versus poorly performing and stable versus unstable cell lines, with the LDA coefficients identifying the key kinetic parameters driving these differences. This is done for two of the CLD campaigns, mAb-A and mAb-B1.

**Fig. 5.**
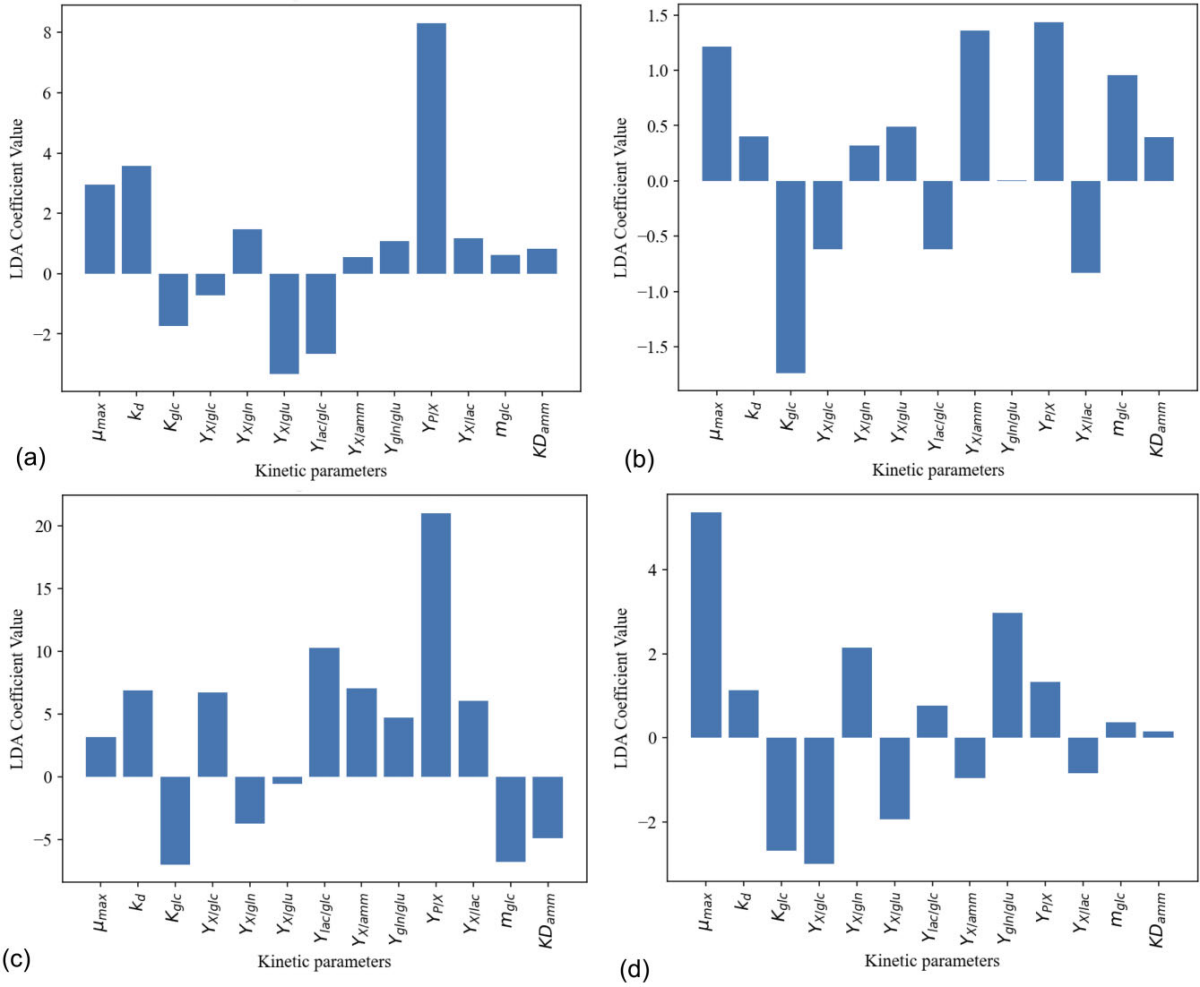
LDA upon the MCKM's regressed kinetic parameters to study the biological differences amongst ‘well performing’ versus ‘poorly performing’ or ‘stable’ versus ‘unstable’ cell lines during CLD. Plotted are the LDA coefficients of the kinetic parameters that distinguish the classes (a) well performing versus poorly performing cell lines of mAb-A campaign (classification accuracy 90%), (b) stable versus unstable cells of the mAb-A campaign (classification accuracy 40%), (c) well performing versus poorly performing cell lines of the mAb-B1 campaign (classification accuracy 75%), (d) stable versus unstable cells of the mAb-B1 campaign (classification accuracy 60%).

The LDA results comparing well- versus poorly performing cell lines producing mAb-A and mAb-B1 are shown in Fig. 5a, c, respectively. Cells that perform ‘well’ were labelled as such, based on scoring top 50% in titre, and having high peak productivity. As expected, *Y_P/X_* provides the strongest discriminatory power between ‘well-’ and ‘poor’ performing cells, reflecting its direct link to productivity and titer. Interestingly, *Y_X/glu_* shows comparable discriminative power as *µ_max_* and *k_d_* for distinguishing ‘well’ performing cells, indicating that cell growth is as critical as the efficiency of glutamate conversion via the GS system for cell line performance. The LDA coefficients for *Y_X/glc_* differ remarably between mAb-A and mAb-B1 (≈ −0.7 vērsus ≈ 7 in Fig. 5a, c, respectively), suggesting that glucose metabolism may be influenced by the type of mAb that is being produced. When producing different antibodies, cells may experience changes in metabolic demands, protein synthesis rates, and cellular stress responses, all of which can influence glucose metabolism (Fan et al., 2015).

When comparing ‘stable’ (i.e. stable productivity over Ambr-1, 2, 3, 4) versus ‘unstable cell’ lines, and the discrimanitory power the kinetic parameters have towards this (Fig. 5b, d for mAb-A and mAb-B1, respectively), *µ_max_, k_D_, K_glc_* and *Y_X/glc_* provide the greatest discriminatory power, highlighting the importance of growth and glucose metabolism in maintaining cell line stability. Efficient conversion of glucose into biomass rather than by-products appears critical for stable performance (Vergara et al., 2018).

The LDA coefficient of *Y_X/amm_* for distinguishing stable versus unstable cell lines, differs between the mAbs: strongly positive for mAb-A, see Fig. 5b, but slightly negative for mAb-B1, Fig. 5d. This suggests that higher ammonium utilization may support stability in mAb-A, whereas lower ammonium utilization may be preferable for mAb-B1. Such differences reflect the known variability in how CHO cells handle metabolic by-products depending on the recombinant protein produced (Noh et al., 2017).

## Conclusions

In this paper, we present the MCKM, a novel generalized kinetic model capable of dynamically simulating a wide range of CHO clonal cell lines and mAb targets. The key innovation of MCKM is its ability to characterize the metabolic kinetics of individual clones derived from the same CHO host, computing a distinct profile of 13 kinetic parameters from a single cell culture run containing only 49 datapoints. This contrasts with most kinetic models in the literature, which typically focus on population-averaged behaviour, require multiple replicate cultures per regression, or aim primarily at production-level fitting rather than capturing clonal heterogeneity. MCKM enables the characterization of individual clones during upstream CLD, providing substantial knowledge gain for cell line selection and for comparing kinetic behaviour across different clones during a CLD campaign, as well as comparing different mAb targets and CHO hosts across campaigns. The model was developed and validated on four historical CLD campaigns, comprising 656 unique fed-batch cell culture regressions (15 mL minibioractor Ambr15™) from 157 unique clones producing three distinct mAbs.

Across 656 regressions, MCKM achieved high accuracy with the average *R^2^* of viable biomass and mAb titre $\bar{R}_{{{X}_v}}^2 \approx $0.96 ± 0.07 and $\bar{R}_P^2 \approx $0.97 ± 0.05 across cell line cultures, respectively, with 99% of runs exceeding *R²* > 0.90. This robustness is remarkable given that the model regresses 13 kinetic parameters from a single cell culture run of only 49 datapoints, whereas most approaches reported in the literature rely on accumulating multiple replicates. The *NRMSE* for biomass (6–7%) and mAb product (4–9%) even outperformed hybrid and transfer models (typically 10–20%).

To achieve such broad applicability across hundreds of clones and three mAb targets, MCKM required unique implementations. One is an automated parameter balancing procedure: if regressions fail due to dimensionality, they are re-run with one growth kinetic parameter fixed from an initial guess, ensuring all 656 runs converge to a biologically interpretable solution. MCKM is novel in its implementation of the lactate switch, implemented as a glucose-limitation function, which successfully captured diverse behaviours—from clones that switch once or twice, to those that continuously produce lactate. Although lactate predictions showed higher error (~50%), the strength of the model lies not in the absolute predictive accuracy, but in providing biologically interpretable parameters that enable cross-clone comparison of lactate metabolism, particularly the frequency and timing of lactate switches. MCKM could be further refined by linking to physiological drivers such as pH, redox balance, or mitochondrial function.

The GS-system was simplified by modelling glutamate and ammonium as functions of growth, allowing MCKM to cover a wide variety of clone-specific profiles. While this abstraction limits accuracy during the death phase, the model still achieved ~10% NRMSE for ammonium and glutamine in two of the four CLD campaigns. Lower *R²* for glucose and glutamate were largely due to estimated feeding times rather than model structure.

A parameter identifiability analysis revealed expected co-linearity among growth kinetics, reflecting underlying biology. Some yields (e.g. glutamine/glutamate, lactate/glucose) were not uniquely identifiable, suggesting that further experimental studies would be required to refine their biological interpretation. Importantly, even with non-unique values, MCKM provides meaningful discrimination when comparing lactate, glutamine, and glutamate profiles across cell lines.

A case study application of MCKM was shown demonstrating its ability to capture kinetic differences between well-performing, stable clones and poorly performing, unstable clones. MCKM could serve as a standard tool in CLD to characterize cell line metabolism, providing additional data for lead clone selection. Beyond this case study, the applications of MCKM are broad. It could predict which clones are likely to remain stable and productive over long culture periods (Dean & Reddy, 2013; Zagari et al., 2013), identify biomarkers or critical growth rates linked to productivity, and guide feeding strategy optimization. Moreover, the model could support metabolic engineering by highlighting genetic or metabolic engineering targets, as well as providing a tool for simulating how clones behave under different conditions in bioreactor conditions.

## Data Availability

The data underlying this article were provided by GlaxoSmithKline under a confidentiality agreement and are available only to the authorized parties in accordance with that agreement.
